# Do lower respiratory tract samples contribute to the assessment of carriage of *Staphylococcus aureus* in patients undergoing mechanical ventilation after major heart surgery?

**DOI:** 10.1371/journal.pone.0207854

**Published:** 2018-12-26

**Authors:** Emilio Bouza, Almudena Burillo, Patricia Munoz, Maricela Valerio, Jose Maria Barrio, Javier Hortal, Gregorio Cuerpo, Maria Jesus Perez-Granda

**Affiliations:** 1 Clinical Microbiology and Infectious Diseases Department, Hospital General Universitario Gregorio Marañón, Madrid, Spain; 2 Instituto de Investigación Sanitaria Gregorio Marañón (IiSGM), Madrid, Spain; 3 Department of Medicine, School of Medicine, Universidad Complutense de Madrid, Madrid, Spain; 4 CIBER de Enfermedades Respiratorias-CIBERES (CB06/06/0058), Madrid, Spain; 5 Cardiac Surgery Postoperative Care Unit, Hospital General Universitario Gregorio Marañón, Madrid, Spain; Hospital Universitari Bellvitge, SPAIN

## Abstract

Colonization by *Staphylococcus aureus* is regularly assessed in patients undergoing major heart surgery (MHS). Despite pre-surgical decontamination attempts, a significant proportion of MHS patients remain colonized by *S*. *aureus* at the time of surgery. Nasal sampling can be improved by sampling extra-nasal areas. We evaluated whether processing lower respiratory tract (LRT) secretions enhanced the detection of *S*. *aureus* after MHS. Following a standard protocol, nasal swabs and LRT aspirates were obtained from all of the study patients at the time of surgery or in the immediate postoperative period. One swab was used for culture in the microbiology laboratory, and a second swab was used for the Xpert SA Nasal Complete assay. According to our definition of colonization (culture positive and/or PCR positive), 31 of 115 patients (26.9%) were colonized at the time of surgery. Among these, LRT samples only were positive in three patients (2.6% of the whole population and 9.7% of the carriers). The remaining 28 were either positive in the nasal sample or positive in both samples. The yield of the detection of colonization by *S*. *aureus* by including also LRT samples in patients undergoing MHS is limited and must be balanced with laboratory workload and demands on laboratory personnel.

**Trial registration**: Clinical trials.gov NCT02640001.

## Background

Nasal carriage of *Staphylococcus aureus* (methicillin-resistant [MRSA] or methicillin-susceptible [MSSA]) prior to surgery is a risk factor for postoperative infection by this microorganism [1–3]. Pre-operative decolonization of nasal carriage of *S*. *aureus* has been shown to be effective in reducing the risk of surgical site infection (SSI), including harvest site and organ/space sternal SSI [4–9]. It is also cost-effective [10,11].

Carriage of *S*. *aureus* is usually determined by bilateral nasal swabbing followed by culture, molecular techniques, or both [12–14].

Our group recently demonstrated that patients undergoing major heart surgery (MHS) are frequently colonized with *S*. *aureus* at the time of surgery or immediately after, even despite previous decolonization attempts (article under submission), as reported elsewhere [8].

Nasal samples from *S*. *aureus* carriers are sometimes negative, although samples from other locations may be positive [15–18]. In a recent article, nasal swabs, pharyngeal swabs, and swabs from the groin were positive in 65.7%, 6.1%, and 6.6% of cases, respectively; the sum of the three anatomical locations, taken simultaneously, amounted to 98.3% [8].

As intensive care unit (ICU) patients are regularly intubated and lower respiratory tract (LRT) secretions are easily available by tracheal aspiration, we investigated the yield of LRT samples for the assessment of carriage of *S*. *aureus* at the time of surgery or immediately after and compared them with detection based on nasal samples. Our objective was to investigate whether the detection of *S*. *aureus* in LRT samples improved that of nasal samples.

## Material and methods

### Hospital setting and patients

Our institution is a general referral hospital with 1,550 beds and approximately 50,000 admissions/year. The MHS department performs about 500 procedures annually.

### Procedure

Ours is a prospective study. Consecutive patients admitted to the MHS Department during the study period (07 July 2015 to 07 April 2016) were enrolled if they consented to participate.

### Sampling

Patients enrolled in the study had nasal and endotracheal aspirates taken in parallel at the time of cardiac surgery or in the immediate postoperative period. Weekly samples continued to be taken in patients who remained intubated.

Two swabs were taken from both anterior nares for each sample (one for culture and another for rapid molecular detection).

Tracheal aspirates were obtained using a Lukens trap, as reported elsewhere [19].

Once taken, both nasal and LRT samples were sent to the microbiology department immediately.

### Laboratory procedure (culture and polymerase chain reaction [PCR])

#### Sample processing

Nasal and endotracheal samples were cultured in the microbiology laboratory. *S*. *aureus* was detected in both samples using molecular methods (Xpert SA Nasal Complete assay). Samples were processed using PCR according to the manufacturer's instructions, as detailed elsewhere [20]. Xpert is a qualitative *in vitro* diagnostic test designed for rapid detection of MSSA and MRSA from nasal swabs. The test utilizes automated real-time PCR to detect nucleic acid sequences of the staphylococcal protein A (*spa*), the gene for methicillin/oxacillin resistance (*mecA*), and staphylococcal cassette chromosome (SCC*mec*).

Samples were cultured on a mannitol-salt agar plate and on a chromogenic medium for the isolation of MRSA (chromID MRSA, bioMérieux, Craponne, France) and processed for a semiquantitative count.

Plates were incubated for 48 hours at room temperature.

### End-points

- The percentage of colonized patients based only on the LRT samples.
- Comparison of the negative yields of culture and the PCR technique in both nasal swabs and endotracheal aspirates.

### Definitions

Colonization was defined as a positive PCR result or a positive culture with *S*. *aureus* in either nasal secretions or endotracheal aspirates.

### Ethics

The local ethics committee (Ethics Committee of Hospital General Universitario Gregorio Marañón, Madrid, Spain) approved the study. Written informed consent was obtained from the study participants.

### Statistical analysis

Continuous variables are expressed as the mean (SD) or median (IQR); categorical variables are expressed as continuous variables and as percentages, with a 95% confidence interval (CI), when applicable. Categorical variables were evaluated using the chi-square test or a 2-tailed Fisher exact test. Statistical significance was set at p<0.05 (2-tailed).

The statistical analysis was performed using IBM SPSS Statistics for Windows, Version 21.0 (IBM Corp, Armonk, New York, USA).

We calculated the validity values of the culture and PCR of nasal swabs and endotracheal aspirates by comparing non-colonized patients with colonized patients. The sensitivity, specificity, and positive and negative predictive values with their 95% CI, were calculated using EPIDAT 3.1.

## Results

**Fig 1:**
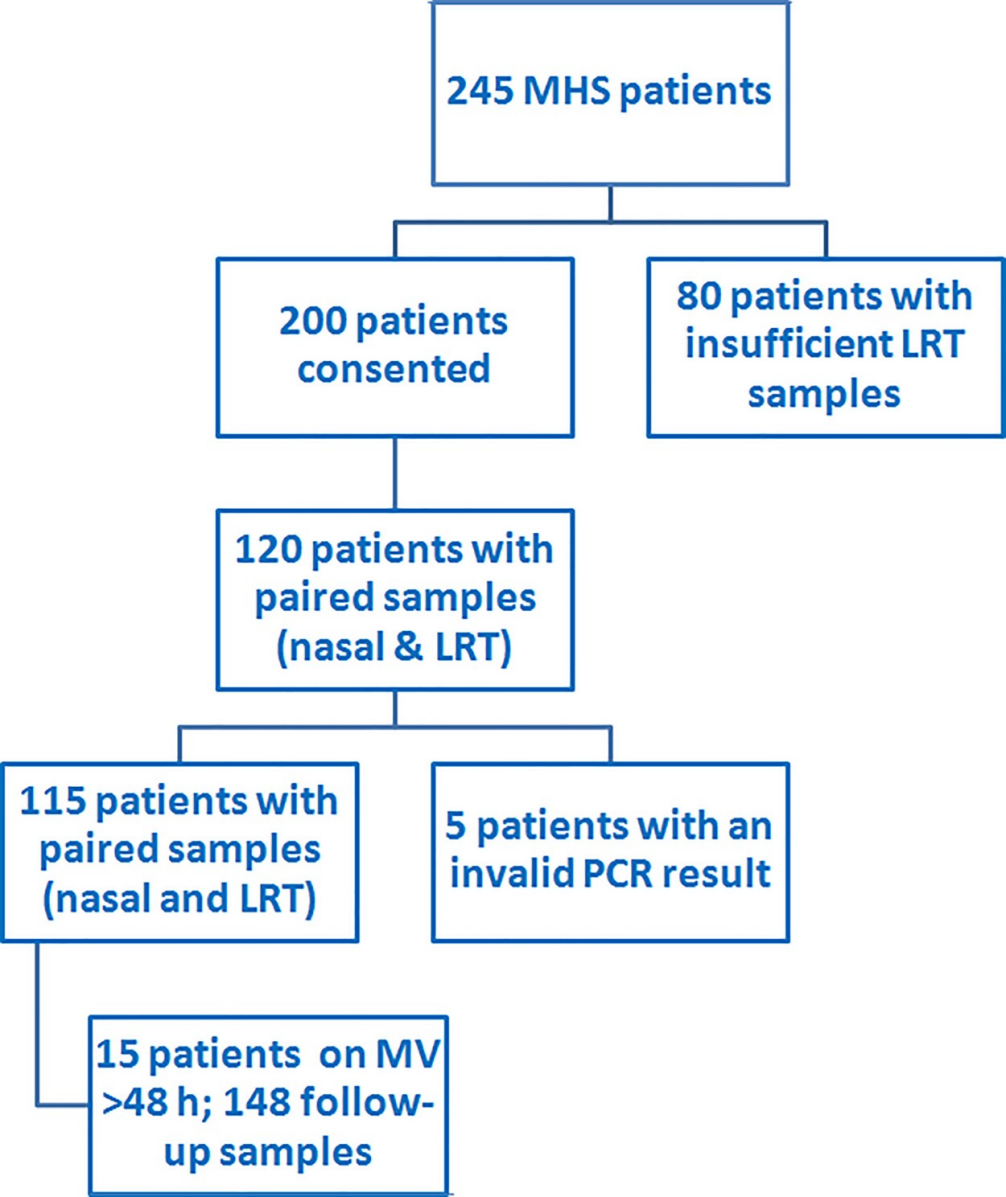


A total of 254 patients underwent MHS during the study period (07 July 2015 to 07 April 2016); of these 200 fulfilled the inclusion criteria. The LRT sample was insufficient for testing in 80 patients (owing to absence of respiratory secretions). In 5 patients, 1 or more of the LRT samples were invalid in the PCR assay. The remaining 115 patients constitute the basis of our study **(Fig 1).** We were able to obtain 1 or more paired follow-up samples in 15 patients who remained intubated. Overall, we obtained 148 paired samples (nasal and LRT).

**Table 1** shows the characteristics of the study population (115 patients). The median (IQR) age was 68 (58–76) years. The main underlying conditions were congestive heart failure, diabetes mellitus, chronic obstructive pulmonary disease (COPD), and myocardial infarction. The main surgical procedure was valve replacement (Table 1). The mean (SD) EuroSCORE and median (IQR) APACHE II score at inclusion were, respectively, 6.5 (3.5) and 9.0 (7–11).

**Table 1:** Patient characteristics.

	Total (n = 115)
**Preoperative**	
Median (IQR) age in years	68 (58.0–76.0)
Male/Female	75/40
**Underlying conditions (%)**	
Myocardial infarction	19 (16.5)
Congestive heart failure	70 (60.9)
Central-nervous system disease	10 (8.7)
Chronic obstructive pulmonary disease	21 (18.3)
Renal dysfunction	13 (11.3)
Diabetes mellitus	34 (29.6)
Peptic ulcer disease	10 (8.7)
Peripheral vascular disease	14 (12.2)
EuroSCORE (±SD)	6.5 (3.5)
Apache II, median (IQR)	9.0 (7.0–11.0)
**Type of surgery (%)**	
Valve replacement	54 (47.0)
CABG	25 (21.7)
Mixed (valve and CABG)	16 (13.9)
Aortic surgery	7.0 (6.1)
**Median hospital stay in days (IQR)**	21 (14.0–33.0)
**Median ICU stay in days (IQR)**	6.0 (4.0–10.0)
**Respiratory infection (%)**	12 (10.4)
**Other infection (%)**	18 (15.7)
**Mortality (%)**	17 (14.8)

Overall, 31/115 patients (26.9%) were colonized according to our definition (culture positive, positive by PCR or both in either nasal or LRT samples at the time of surgery, first sample). Of these, 3 patients were colonized only in the LRT samples (9.7% of the colonized cases and 2.6% of the total population). Seven patients were colonized in both samples (22.6%), and 21 (67.0%) had positive nasal samples only.

**Table 2** shows the results of culture and PCR in both samples at admission. The negative predictive value (NPV) of PCR in nasal and LRT samples was, respectively, 96.6% and 80.0%. However, the NPV of nasal and LRT cultures was 84.0% and 74.3%, respectively.

**Table 2:** Positive first sample, 115 patients.

	SEN^b^ % 95% CI^c^	SPE^d^ % 95% CI^c^	PPV^e^ % 95% CI^c^	NPV^f^ % 95% CI^c^	Validity index 95% CI^c^	Prevalence 95% CI^c^	LR+^g^	LR-^i^
							95% CI^c^	95% CI^c^
**Nasal culture**	48.4	100.0	100.0	84.0	86.1	27.0	ND^h^	0.52
	(29.2–7.6)	(99.4–100.0)	(96.7–100.0)	(76.3–91.7)	(79.3–92.9)	(18.4–35.5)		(0.37–0.73)
**Nasal PCR**^a^	90.3	100.0	100.0	96.6	97.4	27.0	ND^h^	0.10
	(78.3–100)	(99.4–100.0)	(98.2–100.0)	(92.1–100.0)	(94–100.0)	(18.4–35.5)		(0.03–0.28)
**Tracheal aspirate culture**	6.5	100.0	100.0	74.3	74.8	27.0	ND^h^	0.94
	(0–16.7)	(99.4–100.0)	(75.0–100.0)	(65.8–82.8)	(66.4–83.2)	(18.4–35.5)		(0.85–1.03)
**Tracheal aspirate PCR**^a^	32.3	100.0	100.0	80.0	81.7	27.0	ND^h^	0.68
	(14.2–50.3)	(99.4–100.0)	(95.0–100.0)	(71.9–88.1)	(74.2–89.2)	(18.4–35.5)		(0.53–0.86)

^a^PCR: polymerase chain reaction.

^b^SEN: sensitivity.

^c^CI: confidence interval.

^d^SPE: specificity.

^e^PPV: positive predictive value.

^f^NPV: negative predictive value.

^g^LR+: positive likelihood ratio.

^h^ND: not determined (no false positive results).

^i^LR-: negative likelihood ratio.

### Follow-up samples

We were able to obtain follow-up samples (once weekly) from 15 patients who were under mechanical ventilation for over a week. Only 4 patients had 1 or more positive samples. Of these, 3 were already colonized in the first sample. The only patient who was colonized exclusively in the follow-up samples was a patient who proved to be nasal PCR–positive 1 week after the first negative samples.

## Discussion

Our study confirms that, despite decolonization programmes, a high percentage of patients are colonized by *S*. *aureus* at the time of MHS. Only 9.7% of *S*. *aureus* carriers are detected by including LRT secretions as part of screening.

Nasal carriage of *S*. *aureus* is a well-known risk factor for the development of postoperative infections after various surgical procedures, mainly MHS. The morbidity and mortality of those infections are significant [6,21,22]. The nose is unquestionably the best watchtower for surveillance of carriage of *S*. *aureus*, although the yield of detection increases when other samples are taken (e.g., axillary and perineal) [23–25].

Surveillance of *S*. *aureus* in the perioperative period provided us with the opportunity to assess the potential added value of including LRT samples. Our findings show an overall gain of 10.0% for positive patients.

Our data are consistent irrespective of whether the definition of colonization included patients with a positive culture, patients with a positive PCR, or both. In our experience, the gap between PCR results and culture results may have several explanations, including attempts at decolonization before surgery. The fact that we did not have a single case of a patient who was culture-positive and PCR-negative indicates that PCR is very sensitive and fast for daily clinical practice.

In our series, follow-up cultures added only a single patient, who became colonized after initially negative samples. Consequently, we do not recommend continuing surveillance when the patient is in the ICU after admission.

The main limitation of our study is that it was performed only in ICU patients admitted after MHS. However, it is precisely in this group that a higher clinical impact of this measure has been demonstrated [5]. Besides, it was sometimes impossible to obtain LRT secretions from patients intubated at surgery.

The clinical impact of using a rapid PCR technique for the assessment of *S*. *aureus* carriage during MHS procedures, or immediately after, followed by decontamination should be demonstrated in further clinical trials.

We conclude that the yield of detection of *S*. *aureus* by including LRT samples in patients undergoing MHS is limited and must be balanced with workload and demands on laboratory personnel.

## Supporting information

S1 File Checklist. (PDF)

## Abbreviations

MHS: major heart surgery
LRT: lower respiratory tract
PCR: polymerase chain reaction
MSSA: methicillin-susceptible *Staphylococcus aureus*
MRSA: methicillin-resistant *Staphylococcus aureus*
SSI: surgical site infection
COPD: chronic obstructive pulmonary disease
